# Effect of Keishibukuryogan on Endothelial Function in Patients with at Least One Component of the Diagnostic Criteria for Metabolic Syndrome: A Controlled Clinical Trial with Crossover Design

**DOI:** 10.1155/2012/359282

**Published:** 2012-05-22

**Authors:** Yutaka Nagata, Hirozo Goto, Hiroaki Hikiami, Tatsuya Nogami, Makoto Fujimoto, Naotoshi Shibahara, Yutaka Shimada

**Affiliations:** ^1^Department of Japanese Oriental Medicine, Graduate School of Medicine and Pharmaceutical Sciences, University of Toyama, 2630 Sugitani, Toyama 930-0194, Japan; ^2^Division of Kampo Diagnostics, Institute of Natural Medicine, University of Toyama, 2630 Sugitani, Toyama 930-0194, Japan

## Abstract

We evaluated the effect of keishibukuryogan (KBG; Guizhi-Fuling-Wan), a traditional Japanese (Kampo) formula, on endothelial function assessed by reactive hyperemia peripheral arterial tonometry (Endo-PAT2000) in patients with metabolic syndrome-related factors by controlled clinical trial with crossover design. Ninety-two patients were assigned to group A (first KBG-treatment period, then control period; each lasting 4 weeks, with about one-year interval) or group B (first control, then KBG-treatment). In forty-nine (27, group A; 22, group B) patients completing all tests, the mean value of the natural logarithmic-scaled reactive hyperemia index (L_RHI) increased and those of serum nonesterified fatty acid (NEFA), malondialdehyde, and soluble vascular cell adhesion molecule 1 decreased significantly during the KBG-treatment period, but not during the control period, and 4-week changes of L_RHI, NEFA, and malondialdehyde between the 2 periods showed significance. These results suggest that KBG has beneficial effect on endothelial function in patients with metabolic syndrome-related factors.

## 1. Introduction

In Japan, the incidence of cardiovascular events has been increasing on account of the westernization of lifestyle and increases in the prevalence of overweight and metabolic syndrome [1, 2]. Recently, endothelial dysfunction has been recognized as a crucial pathogenesis in the early stage of arteriosclerosis [3, 4]. Traditional risk factors such as hypertension, dyslipidemia, and hyperglycemia are associated with endothelial dysfunction [4], and endothelial dysfunction itself is also reported to be an independent risk factor in the development of cardiovascular events [5]. Endothelial dysfunction is reversible, and its improvement can prevent the development of arteriosclerosis.

 Flow-mediated dilatation (FMD) has been used for the measurement of endothelial function, and by this method the change of forearm vascular diameter is evaluated by ultrasonic apparatus [6]. On the other hand, reactive hyperemia peripheral arterial tonometry (RH-PAT), a noninvasive apparatus developed recently, allows easier measurement compared to FMD [7, 8]. It requires a less-operator-dependent technique, and the influence of sympathetic nervous activity in RH-PAT is less than that in FMD. Therefore, it makes possible the comparison of measured values from different devices.

 Keishibukuryogan (KBG; Guizhi-Fuling-Wan) is a traditional Japanese (Kampo) formula used to prevent the development of atherosclerosis. In recent years, we reported the protective effects of KBG on endothelial function in cholesterol-fed rabbits and spontaneously diabetic rats [9, 10]. Further, we revealed that KBG actually inhibits the progression of atherosclerosis in cholesterol-fed rabbits [11, 12]. However, it has not been assessed whether KBG prevents the progression of atherosclerosis clinically in human subjects, and long-term study is not easy to conduct. Therefore, this time we set out to evaluate the effects of KBG on endothelial function using RH-PAT in patients with metabolic syndrome-related factors by a controlled clinical trial with crossover design.

## 2. Methods

### 2.1. Patients

The diagnostic criteria for metabolic syndrome adopted in the study were defined by the Examination Committee for Criteria of metabolic syndrome in Japan, that is, (1) waist circumference ≥85 cm in men and ≥90 cm in women; in addition 2 or more of the following 3 components: (2) triglyceride (TG) ≥ 150 mg/dL and/or high-density lipoprotein (HDL) cholesterol < 40 mg/dL, (3) systolic blood pressure (SBP) ≥ 130 mmHg and/or diastolic blood pressure (DBP) ≥ 85 mmHg, and (4) fasting plasma glucose (FPG) ≥ 110 mg/dL [13]. We recruited patients aged 40–80 years who were consulting the Department of Japanese Oriental Medicine, Toyama University Hospital, for treatment of various diseases or symptoms, and having 1 or more components of the above diagnostic criteria between June 2008 and August 2011. Patients with serious liver or kidney disease, infectious disease, malignancy, previous stroke or myocardial infarction, or other diseases considered to possibly disturb the implementation of this trial, were excluded from enrollment.

### 2.2. KBG

 KBG consists of 5 dried herbal medicines: Cinnamomi Cortex, Paeoniae Radix, Moutan Cortex, Persicae Semen, and Hoelen. These herbal powders were mixed with boiled honey at the ratio shown in Table 1 and rolled up into balls (2 g each). All these herbal medicines and honey were purchased from Uchida Wakanyaku Co. (Tokyo, Japan).

### 2.3. Study Design

**Figure 1:**
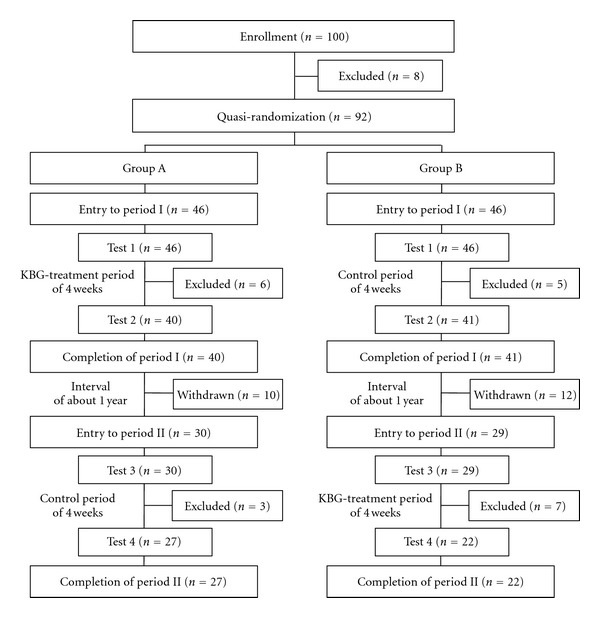
Flow chart of patients enrolled in the study.

 The study was a controlled clinical trial with crossover design consisting of a 4-week KBG-treatment period and a 4-week control period. The patients were randomly assigned into group A (KBG-treatment, period I; control, period II) or group B (control, period I; KBG-treatment, period II). The Framingham heart study using the FMD technique revealed that flow-mediated vascular dilatation is influenced by season or temperature and is highest in summer and lowest in winter [14]. Therefore, we set both period I and period II in the same season, and the interval between the 2 periods at about one year. Patients were evaluated at most 4 times (test 1, beginning of period I; test 2, end of period I; test 3, beginning of period II; test 4, end of period II). KBG (6 g per day) was administered three times a day after meals in addition to their usual prescribed drugs for 4 weeks in period I in group A or period II in group B. These concomitant drugs had not been changed for at least 3 months before the beginning point of and during period I and period II (Figure 1). This study was an open-label study, as it was impossible to prepare a suitable placebo due to the unique flavor and taste of KBG.

 The study design was approved by the Ethics Committee, University of Toyama. All patients provided written informed consent in accordance with the ethical guidelines set forth in the 1975 Declaration of Helsinki.

### 2.4. RH-PAT

**Figure 2:**
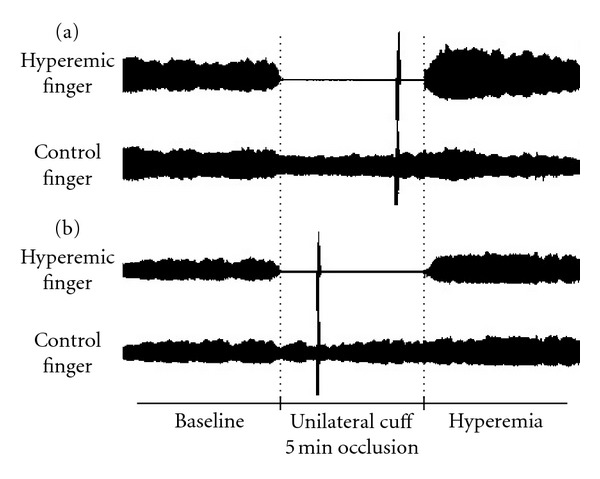
Representative signals of reactive hyperemia peripheral arterial tonometry (RH-PAT) with (a) normal and (b) low-reactive hyperemic response. Normal response characterized by a distinct increase in the signal amplitude after cuff release compared with baseline.

 Endothelial function was evaluated by Endo-PAT2000 (Itamar Medical, Caesarea, Israel). The principle of RH-PAT has been described previously [7, 8, 15]. Briefly, a blood pressure cuff was placed on one upper arm, while the contralateral arm served as control. PAT probes were placed on one finger (finger II or III) of each hand (same finger on both hands). After a 5-minute equilibration period, the cuff was inflated to the higher of either 60 mmHg above systolic pressure or 200 mmHg for 5 min and then deflated to induce reactive hyperemia. PAT signals were recorded electronically in both fingers. During these procedures, the subject was requested to remain quiet and still, and the room temperature was controlled at 21–24°C. The RH-PAT data were analyzed by computer with Endo-PAT2000 software version 3.1.2 in an operator-independent manner. The PH-PAT index reflects the extent of reactive hyperemia and was calculated as the ratio of the average amplitude of PAT signal over 1 min starting 1.5 min after cuff deflation (hyperemic finger, A; control finger, C) divided by the average amplitude of the PAT signal of a 2.5-minute duration before cuff inflation (baseline, hyperemic finger, B; control finger, D). Then, the reactive hyperthermia index (RHI) was obtained from this equation: RHI = (A/B)/(C/D) × baseline correction factor. Recently, the natural logarithmic scaled RHI (L_RHI) has often been used instead of RHI [4, 15, 16], and we also used it in this study (Figure 2).

### 2.5. Physical Findings and Laboratory Data

Immediately before the examination of RH-PAT, patients were evaluated for physical findings, such as height (HT), body weight (BW), waist circumference, and SBP and DBP after 5-minute rest at supine position. Body mass index (BMI) was also calculated (BMI = BW (kg)/HT (m)^2^).

 Blood was collected from the cubital vein after overnight fasting immediately after the RH-PAT examination, and partially separated serum was frozen at −80°C immediately and stored until assay. Routine clinical laboratory data, such as TG, HDL-cholesterol, low-density lipoprotein (LDL) cholesterol, FPG, immunoreactive insulin (IRI), creatinine, and high-sensitive C-reactive protein (hs-CRP), were measured by standard laboratory techniques in our hospital. Homeostasis model assessment as an index of insulin resistance (HOMA-IR) was performed for the evaluation of insulin resistance: [HOMA-IR = IRI (*μ*U/mL) × FPG (mg/dL)/405] [17]. Nonesterified fatty acid (NEFA; free fatty acid) was measured by enzyme method at Mitsubishi Chemical Medience Co., Tokyo, Japan. We measured malondialdehyde (MDA), a marker of oxidative stress, by thiobarbituric acid reactive substance (TBARS) assay kit (OXI-TEK TBARS Assay Kit; ZeptoMetrix Co., New York, USA), and soluble vascular cell adhesion molecule 1 (sVCAM-1) by Human sVCAM-1 Quantikine ELISA Kit (R&D Systems Inc., Minneapolis, USA) in our laboratory.

### 2.6. Statistical Analysis

 Statistical analysis was performed with JMP 9 (SAS Institute Japan, Tokyo). Data were expressed as mean ± S.E. Either Wilcoxon test or Pearson's chi-square test was used for statistical analysis of the patient's characteristics. The difference between the data at week 0 and week 4 was analyzed using the Wilcoxon matched-pairs signed-ranks test. Comparison between the change of values in KBG-treatment period and control period was performed by MANOVA test. A value of *P* < 0.05 was considered statistically significant.

## 3. Results

### 3.1. Patients

 In total, 100 patients were initially enrolled, but 8 were then excluded (6, deviation from inclusion criteria; 2, consent withdrawn), and 92 patients were finally quasi-randomized to group A (*n* = 46) and group B (*n* = 46), and entered into period I (Figure 1).

 In group A, after test 1, 6 patients were excluded [3, refused intake of KBG; 1, adverse effect (glossalgia); 1, change of concomitant drug; 1, eating before test], and after the 4-week KBG-treatment period the remaining 40 patients underwent test 2 and completed period I. During the interval of about 1 year between periods I and II, 10 patients dropped out (7, refused to participate; 2, onset of other disease; 1, discontinued hospital visit). The other 30 patients entered into period II and underwent test 3. After that, 3 patients were excluded (1, onset of infectious disease; 2, change of concomitant drug), and after the 4-week control period, the remaining 27 patients underwent test 4 and completed period II.

 In group B, after test 1, 5 patients were excluded (3, refused to participate; 1, onset of infectious disease; 1, eating before test), and after the 4-week control period, the remaining 41 patients underwent test 2 and completed period I. During the interval between periods I and II, 12 patients dropped out (7, refused to participate; 1, onset of other disease; 4, discontinued hospital visit), and the other 29 patients entered into period II and underwent test 3. After that, 7 patients were excluded (2, adverse effects (diarrhea, mouth bitterness); 4, change of concomitant drug; 1, eating before test), and after the 4-week KBG-treatment period, the remaining 22 patients underwent test 4 and completed period II.

### 3.2. Patient Characteristics

 The baseline characteristics of the patients who completed all the tests are shown in Table 2. A statistical difference between the 2 groups was seen in male waist circumference, but not in any of the other factors.

### 3.3. Effects of KBG

 Comparison between various parameters at week 0 and week 4 in both the KBG-treatment period and control period and that of 4-week changes between the KBG-treatment period and control period in patients completing all tests (49 patients in group A + B, 27 in group A plus 22 in group B) are shown in Table 3. The mean value of L_RHI in the KBG-treatment period increased significantly, but not in the control period, and the 4-week changes between KBG-treatment and control periods showed statistical significance. Further, the mean values of NEFA, MDA, and sVCAM-1 in the KBG-treatment period decreased significantly, and 4-week changes of the former 2 parameters between KBG-treatment and control periods were statistically significant.

 Separate data of group A (Table 4) and group B (Table 5) are also shown. In group A, the mean value of L_RHI in the KBG-treatment period increased significantly, but not in the control period, and the 4-week changes between the KBG-treatment and control periods showed statistical significance. The mean value of NEFA in the KBG-treatment period decreased significantly, and the 4-week changes between the KBG-treatment and control periods showed statistical significance. Further, the levels of MDA and sVCAM-1 in the KBG-treatment period decreased significantly. Similarly in group B, the mean value of L_RHI in the KBG-treatment period increased significantly. The levels of NEFA and MDA in the KBG-treatment period decreased significantly, and the 4-week changes of MDA levels between the KBG-treatment and control periods showed statistical significance.

## 4. Discussion

The risk factors of cardiovascular diseases, such as diabetes mellitus, hypertension, and dyslipidemia, often accumulate in the same person and increase the incidence of cardiovascular events synergistically. The concept of metabolic syndrome arose from a global movement to unify such high-risk pathological conditions. In Japan also, clinical diagnostic criteria of metabolic syndrome were announced in 2005 [13]. Endothelial dysfunction is often seen in patients with metabolic syndrome, and it is recognized as a primary pathogenic factor of atherosclerosis [4, 18]. Metabolic syndrome is an independent risk factor for cardiovascular events, and if it is combined with endothelial dysfunction, the risk is elevated further [19].

 Several methods have been developed to evaluate endothelial function, and they enable us to perform the examination even for outpatients. Evaluation of endothelial function using FMD or PAT is useful for the early detection of arteriosclerosis. The RHI calculated using the PAT signal is applied to a parameter of endothelial function. Endothelial function in the brachial circulation correlates with that in the coronary circulation, and low RHI is useful for identifying a patient with coronary endothelial dysfunction [7]. Thus, PAT is considered to be a useful, noninvasive examination for the prediction of later cardiovascular events [16]. A value of RHI ≤ 1.67 (L_RHI ≤ 0.51) measured by Endo-PAT2000 is considered to be endothelial dysfunction, is determined in the population with a risk for ischemic heart disease, and is recognized as a cut-off value [20]. The relationship between RHI and metabolic syndrome-related components had been reported [4]. A specific characteristic of endothelial function is its reversibility, and it can be a novel therapeutic target. Studies concerning the effect of drug or supplement on endothelial function using RHI have been reported on the basis of its sensitive reaction to treatment [21, 22].

 In the present study, we evaluated the endothelial function of patients with metabolic syndrome-related factors using RHI by controlled clinical trial with crossover design and revealed that KBG has the potential to improve endothelial function. That is, L_RHI increased significantly in the KBG-treatment period, but not in the control period. It was reported that patients with lower RHI had a higher incidence of cardiovascular events during the followup period [16], and improvement of impaired endothelial function is important for the prevention of the development of arteriosclerosis [3]. Therefore, KBG might be useful for preventing the progression of endothelial dysfunction and arteriosclerosis. All the patients enrolled in this study were free of obvious findings of arteriosclerosis, but endothelial dysfunction (L_RHI ≤ 0.51 at test 1) was seen in 37.0% (34/92) of them. Most of the conventional methods for assessing arteriosclerosis evaluate its advanced stage. Endothelial dysfunction is observed even in the initial and early stages of the progression of atherosclerosis. Therefore, an intervention for endothelial dysfunction by KBG in an as yet latent stage of arteriosclerosis is considered useful for the prevention of eventual cardiovascular events.

 We have previously reported that KBG improves microcirculation evaluated by erythrocyte aggregability and deformability in patients with multiple old lacunar infarction [23, 24]. In experimental animal models, we have also demonstrated that KBG has protective effects against endothelial dysfunction in cholesterol-fed rabbits and spontaneously diabetic rats [9, 10], in addition to its inhibitory effect against plaque formation [11, 12]. KBG is composed of 5 herbal medicines: Cinnamomi Cortex, Paeoniae Radix, Moutan Cortex, Persicae Semen, and Hoelen. We have reported that polyphenols of Cinnamomi Cortex and Paeoniae Radix have endothelium-dependent relaxative effects [25, 26]. Cinnamaldehyde contained in Cinnamomi Cortex also has endothelium-dependent relaxation effect [27, 28]. These effects of KBG and composing herbal medicines are assumed to contribute to the efficacy of KBG on endothelial function.

 It is reported that the elevated level of NEFA is related to metabolic syndrome and endothelial dysfunction, and that it is an independent predictive factor for cardiovascular events [29]. In our previous study, KBG decreased the plasma level of NEFA in cholesterol-fed rabbits [30], and in the present clinical trial, NEFA was also decreased. Therefore, KBG seems to have the actual potential to decrease NEFA, and this effect might contribute to the improvement of endothelial function.

 In the present study, the levels of MDA decreased. Oxidative stress has been reported to impair endothelial function and accelerate the progression of atherosclerosis. A highly oxidized condition aggravates endothelial dysfunction and depresses nitric oxide production [31, 32], and drugs having antioxidant activity possess protective effects against endothelial dysfunction. Previous reports have indicated that each component of the diagnostic criteria of metabolic syndrome is individually related to oxidative stress and endothelial dysfunction [33]. We reported the antioxidative effect of KBG, and this effect is beneficial for the prevention of endothelial dysfunction and arteriosclerosis [9–12].

 As for sVCAM-1, in our previous study dealing with rheumatoid arthritis patients, KBG decreased the plasma levels of sVCAM-1 [34]. VCAM-1 is expressed on the impaired endothelium is regulated by various factors such as oxidative stress and cytokines [35, 36] and has been considered to be an important risk factor to the progression of atherosclerosis and cardiovascular events [37]. In the present study, the value of sVCAM-1 was decreased by KBG-treatment in group A. We speculated that the antioxidant effect of KBG might lead to the downregulation of VCAM-1.

 From the results of the present study, it is suggested that KBG has the potential to prevent the progression of endothelial dysfunction and arteriosclerosis by its antioxidative effect, and early detection of endothelial dysfunction with PAT and early treatment with KBG might contribute to the prevention of arteriosclerosis.

## 5. Conclusion

 Our present controlled clinical trial with crossover design revealed that KBG improves endothelial function as evaluated by L_RHI in patients with metabolic syndrome-related factors, suggesting that KBG has beneficial effects against the progression of endothelial dysfunction and arteriosclerosis.

## Figures and Tables

**Table 1:** Herbal medicines composing KBG and their ratio.

	Herbal medicine		Ratio (g)
Cinnamomi Cortex	*Cinnamomum cassia* BLUME	Guizhi	0.2
Paeoniae Radix	*Paeonia lactiflora* PALLAS	Shaoyao	0.2
Moutan Cortex	*Paeonia suffruticosa* ANDREWS	Mudanpi	0.2
Persicae Semen	*Prunus persica* BATSCH	Taoren	0.2
Hoelen	*Poria cocos* WOLF	Fuling	0.2

KBG: keishibukuryogan.

These 5 herbal powders were mixed with boiled honey (1 g) and rolled up into balls (2 g each).

**Table 2:** Patient characteristics.

	Group A (*n* = 27)	Group B (*n* = 22)	*P*-value
Age (year)	63.5 ± 1.6	62.0 ± 1.7	0.3925^a^
Sex (male/female)	11/16	9/13	0.9905^b^
Smoking (yes/no)	2/25	1/21	0.6740^b^
Alcohol intake (yes/no)	12/15	11/11	0.6983^b^
BMI (kg/m^2^)	24.1 ± 0.6	22.8 ± 0.6	0.3149^a^
Waist circumference (cm)			
Male	91.5 ± 3.1	83.0 ± 1.6	0.0435^∗a^
Female	81.6 ± 1.7	82.5 ± 2.7	0.9649^a^
SBP (mmHg)	126.3 ± 3.2	123.8 ± 2.6	0.7096^a^
DBP (mmHg)	76.3 ± 2.5	75.7 ± 2.2	0.5938^a^
TG (mg/dL)	129.2 ± 14.6	128.3 ± 14.7	0.6802^a^
HDL-cholesterol (mg/dL)	65.4 ± 3.8	60.4 ± 3.2	0.2515^a^
FPG (mg/dL)	103.7 ± 3.3	101.4 ± 2.6	0.9278^a^
Central obesity (yes/no)	11/16	7/15	0.5193^b^
High blood pressure (yes/no)	16/11	13/9	0.9905^b^
Dyslipidemia (yes/no)	12/15	11/11	0.6983^b^
Hyperglycemia (yes/no)	8/19	7/15	0.8687^b^
Concomitant drugs			
Calcium channel blocker (yes/no)	8/19	7/15	0.8687^b^
ARB or ACE inhibitor (yes/no)	3/24	4/18	0.4817^b^
*α* or *β* blocker (yes/no)	3/24	1/21	0.4038^b^
Statin (yes/no)	11/16	8/14	0.7544^b^
Fibrate (yes/no)	1/26	0/22	0.3618^b^
Sulfonylurea (yes/no)	3/24	1/21	0.4038^b^
Thiazolidine analog (yes/no)	3/24	0/22	0.1066^b^
*α*-glucosidase inhibitor (yes/no)	2/25	1/21	0.6777^b^
Antiplatelet drug (yes/no)	1/26	0/22	0.3618^b^
Season test 1 performed	6/14/5/2	9/5/8/0	0.0682^b^
(Spring/Summer/Fall/Winter)			

BMI: body mass index; SBP: systolic blood pressure; DBP: diastolic blood pressure; TG: triglyceride; HDL: high-density lipoprotein; FPG: fasting plasma glucose; central obesity, waist circumference ≥ 85 cm (male), ≥90 cm (female); high blood pressure, SBP ≥ 130 mmHg and/or DBP ≥ 85 mmHg; dyslipidemia, TG ≥ 150 mg/dL and/or HDL cholesterol < 40 mg/dL; hyperglycemia, FPG ≥ 110 mg/dL; ARB: angiotensin II receptor blocker; ACE: angiotensin-converting enzyme; Spring: March 21 to June 20; Summer: June 21 to September 21; Fall: September 21 to December 20; Winter: December 21 to March 20.

^a^Comparison between group A and group B by Wilcoxon test.

^b^Comparison between group A and group B by Pearson's chi-square test.

Data are expressed as mean ± S.E., *P < 0.05.

**Table 3:** Effects of KBG on various parameters in A + B group (*n* = 49).

	Period	Week 0	Week 4	*P*-value^a^	*P*-value^b^
BMI (kg/m^2^)	KBG	23.5 ± 0.4	23.5 ± 0.5	0.3976	0.3034
	Control	23.5 ± 0.5	23.5 ± 0.5	0.9700	
Waist circumference (cm)	KBG	85.0 ± 1.2	85.0 ± 1.2	0.9280	0.9136
	Control	85.2 ± 1.3	85.3 ± 1.3	0.9462	
SBP (mmHg)	KBG	125.0 ± 2.3	121.0 ± 2.1	0.0321*	0.2591
	Control	123.1 ± 1.9	122.0 ± 1.9	0.3629	
DBP (mmHg)	KBG	74.6 ± 1.6	73.9 ± 1.4	0.1297	0.4088
	Control	73.2 ± 1.4	74.1 ± 1.2	0.5626	
L_RHI	KBG	0.58 ± 0.03	0.70 ± 0.04	0.0003**	0.0034**
	Control	0.64 ± 0.04	0.60 ± 0.03	0.7279	
TG (mg/dL)	KBG	124.3 ± 8.9	123.2 ± 7.8	0.9453	0.5339
	Control	125.6 ± 8.9	119.1 ± 6.2	0.6347	
HDL-cholesterol (mg/dL)	KBG	62.0 ± 2.6	62.0 ± 2.5	0.6699	0.3016
	Control	62.6 ± 2.1	61.4 ± 2.0	0.1810	
LDL-cholesterol (mg/dL)	KBG	132.7 ± 4.8	133.8 ± 5.0	0.5803	0.1155
	Control	131.4 ± 4.6	127.7 ± 5.0	0.0435*	
NEFA (*μ*Eq/L)	KBG	532.9 ± 32.9	450.9 ± 26.0	0.0024**	0.0113*
	Control	507.6 ± 31.9	529.6 ± 29.0	0.3273	
FPG (mg/dL)	KBG	101.0 ± 2.3	101.5 ± 2.8	0.9159	0.2087
	Control	105.5 ± 3.0	103.1 ± 3.1	0.1297	
IRI (*μ*U/mL)	KBG	5.73 ± 0.42	5.47 ± 0.39	0.2609	0.5396
	Control	6.26 ± 0.52	6.26 ± 0.52	0.9156	
HOMA-IR	KBG	1.46 ± 0.12	1.42 ± 0.12	0.3842	0.8010
	Control	1.69 ± 0.17	1.68 ± 0.19	0.8639	
Creatinine (mg/dL)	KBG	0.71 ± 0.02	0.72 ± 0.03	0.8238	0.6513
	Control	0.71 ± 0.02	0.71 ± 0.02	1.0000	
hs-CRP (mg/dL)	KBG	0.13 ± 0.05	0.13 ± 0.04	0.3721	0.7501
	Control	0.11 ± 0.03	0.10 ± 0.02	0.3173	
MDA (nmol/mL)	KBG	11.8 ± 1.1	9.5 ± 1.0	<0.0001**	0.0424*
	Control	12.9 ± 1.9	12.3 ± 1.9	0.2804	
sVCAM-1 (ng/mL)	KBG	723.1 ± 43.4	677.7 ± 40.9	0.0126*	0.1659
	Control	724.3 ± 36.0	714.0 ± 36.3	0.8107	

KBG: keishibukuryogan; BMI: body mass index; SBP: systolic blood pressure; DBP: diastolic blood pressure; L_RHI: natural logarithmic scaled reactive hyperemia; TG: triglyceride; HDL: high-density lipoprotein; LDL: low-density lipoprotein; NEFA: nonesterified fatty acid; FPG: fasting plasma glucose; IRI: immunoreactive insulin; HOMA-IR: homeostasis model assessment as an index of insulin resistance; hs-CRP: high-sensitive C-reactive protein; MDA: malondialdehyde; sVCAM-1: soluble vascular cell adhesion molecule 1.

^a^Comparison between week 0 and week 4 by Wilcoxon matched-pairs signed-ranks test.

^b^Comparison of 4-week changes between KBG-treatment period and control period by MANOVA test.

Data are expressed as mean ± S.E., *P < 0.05, **P < 0.01.

**Table 4:** Effects of KBG on various parameters in group A (*n* = 27).

	Period	Week 0	Week 4	*P*-value^a^	*P*-value^b^
BMI (kg/m^2^)	KBG	24.1 ± 0.6	24.2 ± 0.6	0.3362	0.6182
	Control	24.2 ± 0.7	24.2 ± 0.7	0.5916	
Waist circumference (cm)	KBG	85.6 ± 1.8	85.8 ± 2.0	0.6699	0.9871
	Control	87.2 ± 1.9	87.3 ± 1.9	0.8962	
SBP (mmHg)	KBG	126.3 ± 3.2	125.4 ± 3.2	0.6820	0.9476
	Control	122.6 ± 2.8	121.5 ± 2.8	0.5591	
DBP (mmHg)	KBG	76.3 ± 2.5	75.9 ± 2.0	0.3790	0.4292
	Control	71.2 ± 1.8	73.3 ± 1.7	0.3028	
L_RHI	KBG	0.58 ± 0.04	0.70 ± 0.05	0.0231*	0.0085**
	Control	0.65 ± 0.05	0.55 ± 0.04	0.1834	
TG (mg/dL)	KBG	129.2 ± 14.6	128.1 ± 12.7	0.7791	0.5937
	Control	123.4 ± 11.0	116.6 ± 8.9	0.5277	
HDL-cholesterol (mg/dL)	KBG	65.4 ± 3.8	66.0 ± 3.6	0.8341	0.6859
	Control	64.5 ± 2.7	64.5 ± 2.4	0.8432	
LDL-cholesterol (mg/dL)	KBG	138.5 ± 5.9	143.1 ± 6.8	0.2268	0.0715
	Control	130.5 ± 5.3	127.3 ± 5.5	0.1422	
NEFA (*μ*Eq/L)	KBG	527.8 ± 49.1	445.4 ± 33.9	0.0306*	0.0004**
	Control	440.0 ± 30.3	545.9 ± 42.4	0.0028**	
FPG (mg/dL)	KBG	103.7 ± 3.3	105.9 ± 4.6	0.3768	0.2550
	Control	108.9 ± 5.0	106.6 ± 5.2	0.7477	
IRI (*μ*U/mL)	KBG	6.71 ± 0.58	6.05 ± 0.58	0.0593	0.1223
	Control	6.86 ± 0.78	7.18 ± 0.79	0.5777	
HOMA-IR	KBG	1.73 ± 0.17	1.63 ± 0.19	0.2001	0.3213
	Control	1.91 ± 0.26	2.00 ± 0.30	0.4815	
Creatinine (mg/dL)	KBG	0.70 ± 0.03	0.70 ± 0.03	0.5742	1.0000
	Control	0.71 ± 0.03	0.71 ± 0.04	0.9734	
hs-CRP (mg/dL)	KBG	0.10 ± 0.03	0.10 ± 0.02	0.1962	0.2681
	Control	0.07 ± 0.01	0.11 ± 0.02	0.0423*	
MDA (nmol/mL)	KBG	10.2 ± 1.5	8.7 ± 1.4	0.0007**	0.5068
	Control	12.8 ± 2.5	11.9 ± 2.1	0.3444	
sVCAM-1 (ng/mL)	KBG	769.5 ± 72.6	708.2 ± 66.3	0.0121*	0.2791
	Control	735.8 ± 51.3	711.6 ± 48.3	0.7389	

KBG: keishibukuryogan; BMI: body mass index; SBP: systolic blood pressure; DBP: diastolic blood pressure; L_RHI, natural logarithmic scaled reactive hyperemia; TG: triglyceride; HDL: high-density lipoprotein; LDL: low-density lipoprotein; NEFA: nonesterified fatty acid; FPG: fasting plasma glucose; IRI: immunoreactive insulin; HOMA-IR: homeostasis model assessment as an index of insulin resistance; hs-CRP: high-sensitive C-reactive protein; MDA: malondialdehyde; sVCAM-1: soluble vascular cell adhesion molecule 1.

^a^Comparison between week 0 and week 4 by Wilcoxon signed-rank test.

^b^Comparison of 4-week changes between KBG-treatment period and control period by MANOVA test.

Data are expressed as mean ± S.E., *P < 0.05, **P < 0.01.

**Table 5:** Effects of KBG on various parameters in group B (*n* = 22).

	Period	Week 0	Week 4	*P*-value^a^	*P*-value^b^
BMI (kg/m^2^)	Control	22.8 ± 0.6	22.7 ± 0.6	0.4477	0.3443
	KBG	22.7 ± 0.6	22.7 ± 0.6	0.7660	
Waist circumference (cm)	Control	82.7 ± 1.7	82.8 ± 1.6	0.9129	0.8807
	KBG	84.1 ± 1.5	84.1 ± 1.3	0.8125	
SBP (mmHg)	Control	123.8 ± 2.6	122.5 ± 2.3	0.4676	0.0808
	KBG	123.4 ± 3.3	115.5 ± 2.2	0.0037**	
DBP (mmHg)	Control	75.7 ± 2.2	75.2 ± 1.6	0.5524	0.7949
	KBG	72.5 ± 1.8	71.5 ± 1.9	0.1567	
L_RHI	Control	0.62 ± 0.05	0.66 ± 0.05	0.2870	0.1751
	KBG	0.56 ± 0.05	0.70 ± 0.05	0.0074**	
TG (mg/dL)	Control	128.3 ± 14.7	122.2 ± 8.6	0.9750	0.7320
	KBG	118.4 ± 8.4	117.2 ± 7.8	0.9251	
HDL-cholesterol (mg/dL)	Control	60.4 ± 3.2	57.7 ± 3.1	0.0718	0.3085
	KBG	57.8 ± 3.2	57.2 ± 3.3	0.4357	
LDL-cholesterol (mg/dL)	Control	132.6 ± 8.0	128.2 ± 9.0	0.1619	0.7604
	KBG	125.5 ± 7.7	122.5 ± 6.7	0.5393	
NEFA (*μ*Eq/L)	Control	590.5 ± 56.6	509.5 ± 39.0	0.1289	0.9939
	KBG	539.1 ± 42.8	457.7 ± 41.0	0.0312*	
FPG (mg/dL)	Control	101.4 ± 2.6	98.8 ± 2.5	0.0881	0.6065
	KBG	97.6 ± 2.9	96.1 ± 2.7	0.1706	
IRI (*μ*U/mL)	Control	5.51 ± 0.63	5.12 ± 0.59	0.1525	0.2374
	KBG	4.53 ± 0.50	4.76 ± 0.44	0.6260	
HOMA-IR	Control	1.42 ± 0.19	1.28 ± 0.17	0.1342	0.2206
	KBG	1.12 ± 0.14	1.15 ± 0.12	0.8140	
Creatinine (mg/dL)	Control	0.71 ± 0.03	0.70 ± 0.03	1.0000	0.4789
	KBG	0.74 ± 0.04	0.75 ± 0.04	0.7539	
hs-CRP (mg/dL)	Control	0.16 ± 0.07	0.09 ± 0.04	0.4895	0.4382
	KBG	0.17 ± 0.10	0.16 ± 0.07	0.8877	
MDA (nmol/mL)	Control	13.1 ± 2.8	12.8 ± 3.3	0.6747	0.0464*
	KBG	13.7 ± 1.7	10.5 ± 1.4	<0.0001**	
sVCAM-1 (ng/mL)	Control	710.1 ± 50.7	716.9 ± 56.4	0.4684	0.3898
	KBG	666.1 ± 36.3	640.3 ± 41.4	0.3021	

KBG: keishibukuryogan; BMI: body mass index; SBP: systolic blood pressure; DBP: diastolic blood pressure; L_RHI: natural logarithmic scaled reactive hyperemia; TG: triglyceride; HDL: high-density lipoprotein; LDL: low-density lipoprotein; NEFA, nonesterified fatty acid; FPG: fasting plasma glucose; IRI: immunoreactive insulin; HOMA-IR: homeostasis model assessment as an index of insulin resistance; hs-CRP: high-sensitive C-reactive protein; MDA: malondialdehyde; sVCAM-1: soluble vascular cell adhesion molecule 1.

^a^Comparison between week 0 and week 4 by Wilcoxon matched-pairs signed-ranks test.

^b^Comparison of 4-week changes between control period and KBG-treatment period by MANOVA test.

Data are expressed as mean ± S.E., *P < 0.05, **P < 0.01.
